# Antenatal Magnesium Sulfate Benefits Female Preterm Infants but Results in Poor Male Outcomes

**DOI:** 10.3390/ph17020218

**Published:** 2024-02-07

**Authors:** Ruth M. McLeod, Ted S. Rosenkrantz, R. Holly Fitch

**Affiliations:** 1 Department of Psychology, College of the Holy Cross, Worcester, MA 01610, USA; 2 Division of Neonatal-Perinatal Medicine, Department of Pediatrics, University of Connecticut School of Medicine, Farmington, CT 06030, USA; rosenkrant@uchc.edu; 3 Department of Psychological Sciences, Behavioral Neuroscience Division, University of Connecticut, Storrs, CT 06269, USA; roslyn.h.fitch@uconn.edu

**Keywords:** magnesium sulfate, prematurity, neuroprotection, sex differences

## Abstract

Magnesium sulfate (MagSul) is used clinically to prevent eclamptic seizures during pregnancy and as a tocolytic for preterm labor. More recently, it has been implicated as offering neural protection in utero for at-risk infants. However, evidence is mixed. Some studies found that MagSul reduced the incidence of cerebral palsy (CP) but did not improve other measures of neurologic function. Others did not find any improvement in outcomes. Inconsistencies in the literature may reflect the fact that sex effects are largely ignored, despite evidence that MagSul shows sex effects in animal models of neonatal brain injury. The current study used retrospective infant data to assess differences in developmental outcomes as a function of sex and MagSul treatment. We found that on 18-month neurodevelopmental cognitive and language measures, preterm males treated with MagSul (n = 209) had significantly worse scores than their untreated counterparts (n = 135; *p* < 0.05). Female preterm infants treated with MagSul (n = 220), on the other hand, showed a cognitive benefit relative to untreated females (n = 123; *p* < 0.05). No significant effects of MagSul were seen among females on language (*p* > 0.05). These results have tremendous implications for risk–benefit considerations in the ongoing use of MagSul and may explain why benefits have been hard to identify in clinical trials when sex is not considered.

## 1. Introduction

Magnesium sulfate (MagSul) is used to prevent eclamptic seizures in preeclamptic mothers, as a tocolytic to slow preterm labor, and to help protect the brains of infants at risk of preterm birth [1,2]. Though shown to be very beneficial for mothers with preeclampsia, evidence of infant benefit is mixed [3]. Some studies show broad protective effects of MagSul treatment, whereas others show no or limited benefits [3].

**Figure 1:**
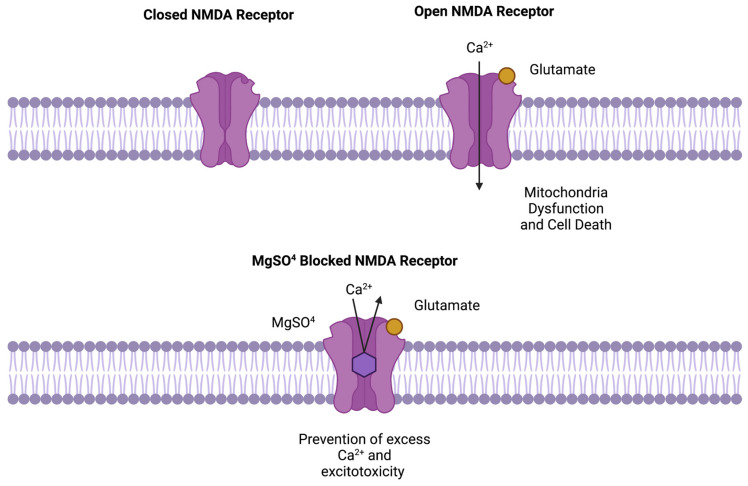
An illustration of magnesium sulfate’s action at the N-methyl-d-aspartate (NMDA) receptor. Top left shows a closed NMDA receptor; top right shows an NMDA receptor opened via extra-cellular glutamate, allowing calcium (Ca^2+^) to enter the cell. Ca^2+^ influx can lead to over excitation, mitochondrial dysfunction, and cell death. Bottom shows MagSul (MgSO^4^) blocking the calcium channel of an open NMDA receptor, thus preventing excitatory glutamatergic effects. *Created with BioRender.com. Accessed 30 January 2024*.

MagSul is a non-competitive N-methyl-d-aspartate (NMDA) receptor antagonist that attenuates cellular calcium influx, unbinding of intracellular calcium, and excitation by blocking the associated ion channel [4] (Figure 1). Based on animal models of induced hypoxic-ischemic brain injury, the mechanism of action provides neuroprotection and reduces apoptosis by suppressing the over-excitation of neurons following oxygen loss, inflammation, or other forms of injury [5,6,7,8,9]. This over-excitation of neurons due to the influx of Ca^2+^ is a major driver of neuronal damage and death following hypoxic injuries, with preterm brains at particular risk due to an over-expression of the NMDA receptor as compared to adults [9]. In guinea pigs, the NMDA receptor expression was found to increase during the second half of gestation leading up to term delivery, in accord with the increased risk of over-excitation in premature infants [10]. Additionally, following hypoxia NMDA receptors are more sensitive to activation, with potential to drive even more excitation [11]. Inflammation can also have a robust detrimental effect on brain development, and both perinatally and in adulthood is associated with poor outcomes following brain injury [12,13,14]. MagSul treatment has not been found to directly reduce inflammation or inflammatory markers, yet does offer neuroprotection from brain injury associated with high levels of brain inflammation [7]. 

In the human literature, putative neuroprotective benefits are less clear. In one clinical trial (*Beneficial Effects of Antenatal Magnesium Sulfate*; BEAM), perinatal MagSul was shown to offer some protection from CP but it did not reduce the risk of moderate to severe CP or death [15]. In another study of infants exposed to chorioamnionitis, maternal MagSul treatment was not found to provide any benefits [16]. Other studies using measures of school-age outcomes showed that MagSul treatment had no impact on behavioral or cognitive outcomes, though treatment did reduce the risk of mortality [17]. In another meta-analysis of the effects of MagSul on infant outcomes, no significant conclusions could be drawn regarding benefits or detriments [18]. The randomized control trials that were included in this meta-analysis showed no difference in reduction of mortality, but also no clear adverse outcomes from MagSul usage, compared to placebos [18]. However, there were possible adverse outcomes identified in non-randomized studies that could require further consideration. For example, there was an increased risk of early germinal matrix or intraventricular hemorrhage (GM/IVH) [18], as originally reported by Salafia [19] in 1995. These investigators found that, along with other factors (e.g., inflammation), MagSul use to delay preterm birth was associated with greater risk of GM/IVH [19]. These mixed results indicate a need to examine the use of MagSul in pregnant mothers more closely, and to examine infant outcomes beyond mortality and brain injury alone.

Neonatal sex differences have rarely been examined in human clinical studies, despite numerous animal studies indicating broad neurodevelopmental sex differences [20,21,22,23,24,25,26]. Critical findings include differential outcomes following HI, as well as sex-specific responses to neonatal neuroprotective treatments [24,25,26,27,28,29]. Moreover, evidence suggests that in some cases the sexes may exhibit comparable neuroprotective benefits despite evidence of different underlying mechanisms of action [30]. For example, the NMDA receptor has shown sex-specific effects following treatment with glucocorticoids used in preterm birth, with dexamethasone showing decreased NMDA expression in females but not in males [31] With specific regard to MagSul, sheep models of perinatal asphyxia revealed that males and females showed some protection from MagSul, but significant sex differences were observed in neural and cardiovascular performance and recovery time [20]. In addition, MagSul reduced asphyxia-related seizures more effectively in males as compared to females, though males had more seizures to begin with [21]. When MagSul is used as a neuroprotective treatment for neuroinflammation in utero, males showed poorer outcomes than females, and thus a greater benefit from treatment [32]. Females in this study did not show much benefit from MagSul treatment, likely related to fewer effects from the inflammation [32]. Of specific interest, in a behavioral study of mice with induced HI, treated subjects showed neuroprotective and anti-inflammatory effects along with reduced tissue loss due to MagSul treatment [33]. However, only female mice showed improved adolescent behavioral outcomes from MagSul treatment [33]. This animal data—combined with the lack of studies considering sex differences in the human MagSul literature—could explain why antenatal MagSul treatment effects have been difficult to characterize. 

The retrospective human infant dataset used in the current study was originally designed to assess the effects of adenosine antagonist (AA) treatment timing (caffeine, theophylline) and AA’s effects on cognitive and language outcomes [34]. For the current study, we examined additional data that had been collected, including sex, prenatal conditions (preeclampsia, y/n), and antenatal MagSul exposure (y/n). Outcome measures included cognitive and language measures at 18 months. We hypothesized possible sex differences following MagSul treatment with respect to these outcomes. 

## 2. Results

Initial analyses were performed on health indices including gestational age (GA), Apgar score, and length of stay for infants that did or did not receive MagSul. Findings confirmed that there were no *a priori* group differences on any of these measures (mean GA’s: MagSul = 27.13, no MagSul = 27.42; Table 1). We performed similar analyses on various health indices as a function of Sex. There was only one significant *a priori* group difference, with female infants having lower birth weights than males (F(1, 184) = 6.112, *p* = 0.014; Table 2). All other health indices were comparable (*p* > 0.05; Table 2). There was no significant relationship seen between sex and cause of premature delivery, either preeclampsia (*X*^2^ (1, 696) = 2.69, *p* = 0.101) or premature rupture of membranes (*X*^2^ (1, 696) = 0.276, *p* = 0.559) (Table 3). There were also no significant interactions between AA (adenosine antagonist) treatment (caffeine or theophylline) and MagSul treatment (*p* > 0.05), either overall or within sexes, on cognitive or language outcomes. 

**Figure 2:**
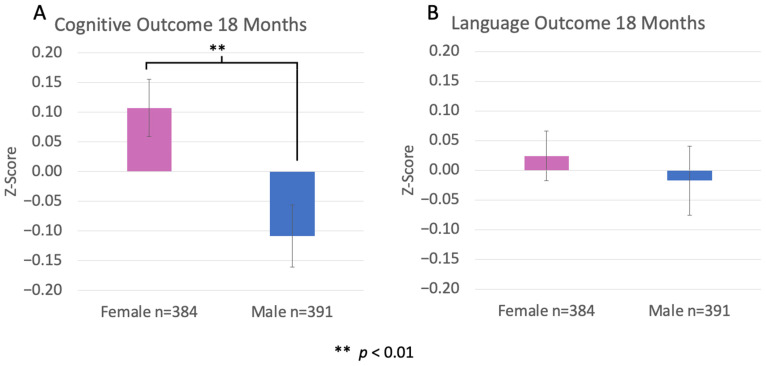
Sex difference in cognitive and language outcomes at 18 months. We report a significant difference between males and females in cognitive performance ((**A**), *p* < 0.01), with females showing better scores. This difference was not significant for language ((**B**), *p* > 0.05). *Adapted with permission from* [34].

For the whole study group, we found a significant main effect of sex on cognitive outcomes (F(1, 775) = 9.114, *p* = 0.003), indicating that preterm females performed better overall cognitively at 18 months as compared to preterm male counterparts (Figure 2A). This difference was not significant for language outcomes (F(1, 775) = 0.335, *p* = 0.563; see Figure 2B; *adapted from* [34]).

**Figure 3:**
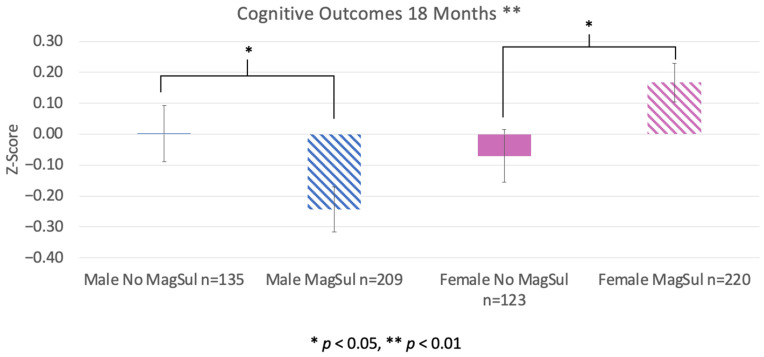
Male and female outcomes as a function of MagSul (magnesium sulfate) treatment. There was an overall significant interaction between MagSul and sex (*p* < 0.01). There was a MagSul effect for males (*p* < 0.05), with MagSul-treated males having a significantly *lower* cognitive performance at 18 months. Females treated with MagSul, on the other hand, performed significantly *better* than untreated (no MagSul) females (*p* < 0.05). Decade and preeclampsia were included as covariates.

We found a significant interaction between sex and MagSul treatment on cognitive outcomes (F(1, 687) = 9.468, *p* = 0.002). Further analysis (simple effects ANCOVA run separately for each sex) showed that males treated with MagSul had significantly lower cognitive scores than untreated male counterparts (F(1, 344) = 4.178, *p* = 0.042). Females showed a significant difference in the opposite direction; MagSul-treated females had significantly higher cognitive scores compared to untreated females F(1, 343) = 4.734, *p* = 0.03, Figure 3). All analyses included decade of birth and preeclampsia as covariates. 

**Figure 4:**
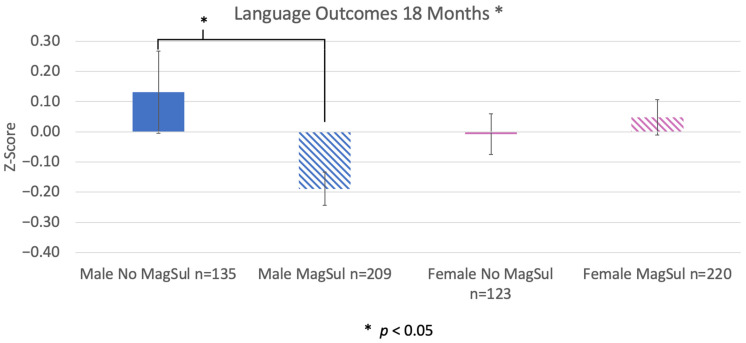
Differences between sexes for MagSul (magnesium sulfate) treated vs. untreated (no MagSul) on language outcomes at 18 months. There was an overall significant difference between all groups (*p* < 0.05). We further saw a significant difference for MagSul-treated males vs. untreated (*p* < 0.05), with treated males having a significantly lower cognitive performance at 18 months. A similar difference was not seen for females. Decade and preeclampsia were run as covariates.

We found a similar interaction between sex and MagSul treatment on language outcomes (F(1, 687) = 5.364, *p* = 0.021). Again, simple effects ANCOVA run separately for each sex showed that males treated with MagSul had significantly lower language scores than untreated counterparts (F(1, 344) = 6.54, *p* = 0.011), while MagSul-treated females showed an opposite pattern (higher mean outcomes, although the difference was not significant, F(1, 343) = 0.237, *p* = 0.627, Figure 4). Again, analyses were run with decade of birth and preeclampsia as covariates. 

## 3. Discussion

Our current results draw renewed attention to the clinical relevance of sex differences in evaluating treatment efficacy, specifically within the newborn population where sex is often ignored. Our findings are consistent with sex differences reported in animal studies, including evidence that females show better outcomes than males in animal models of prematurity and early brain injury [20,21,22,23,25]. A key novel finding is the interaction between sex and MagSul treatment for 18-month outcomes. We found that males treated antenatally with MagSul showed significantly worse cognitive and language outcomes, while females showed a significant cognitive benefit as assessed at 18 months. With regard to potential confounds, we did not see any interaction between MagSul effects and concomitant AA treatment, nor any other medical/health variables assessed. Although we did see an initial significant difference in birthweight between males and females (as expected, given a population mean newborn sex weight difference of 4 ounces), any associated underlying medical condition related to reduced weight would be expected to reflect poorer health and thus would be expected to bias our results in a direction opposite to that observed (with females performing better overall; Figure 2). 

One possible explanation for the robust sex difference in response to MagSul could relate to greater reduction in excitotoxic neural activity in treated preterm females [20]. This could relate to well-established sex differences in caspase-dependent or independent cascades of excitotoxicity and cell death, which have been documented in both adult and neonatal animal models [35,36,37,38]. Specifically, it is possible that MagSul could selectively reduce deleterious excitotoxicity more effectively in females (who show more caspase-dependent cell death following insult) and could enhance deleterious excitotoxicity in males (who show elevated activation of caspase-*independent* cell death cascades [35,36,37,38]). Sex differences in response to MagSul treatment could also reflect evidence that the development and function of the NMDA receptor and ion channel are rapidly changing in the fetus during the last trimester of pregnancy and follow sexually dimorphic trajectories [39,40,41,42]. Moreover, infant male brains show more excitatory NMDA receptors overall, making them more vulnerable to hypoxia and other events that activate the NMDA receptor [43]. Unfortunately, mechanistic studies of developmental sex differences in NMDA receptor maturation remain sparse. There is also evidence from rodent studies that in the absence of injury, MagSul may in general cause enhanced cell death and reduced plasticity [44,45]. This may mean that in cases of low risk of infant injury, MagSul may cause more harm than benefit, especially for males. This, in turn, could relate to significantly elevated levels of circulating androgen in the third trimester male fetus, since androgens have been directly associated under specific conditions with enhanced excitotoxicity and cell death [22].

Finally, in addition to the above, there is also evidence of differential sex effects of MagSul on the placenta, with fetal females showing more vasodilation of placental vessels, potentially leading to different amounts of fetal nutrition and oxygen delivered to the fetus [46]. 

The current findings have tremendous clinical implications given the broad perinatal use of MagSul as a neuroprotectant and the widely accepted assumption of minimal deleterious side effects in the setting of expected preterm labor and delivery. Specifically, the underlying and widely accepted assumption among neonatal medical staff that MagSul provides newborn protection—even in the face of failure to deter labor—contributes heavily to the use of MagSul in otherwise marginal situations as well as an outright prophylactic use of MagSul in preterm laboring mothers [47]. Our results call this assumption pointedly into question. Moreover, mothers receiving MagSul are likely to experience side effects that cause discomfort [2], and high doses of MagSul increase the risk of even more severe side effects [47]. Thus, the routine use of MagSul where pre-eclampsia is *not* a risk may not be beneficial to the fetus or mother. As such, we argue that clinicians need to critically re-evaluate risk-benefit analyses regarding the administration of MagSul to pregnant women.

More broadly, it seems likely that the sex differences reported here explain the mixed results regarding MagSul treatment and infant outcomes to date. Reporting grouped male and female outcomes would logically lead to very mixed findings depending on subject pool composition. Thus our findings further draw renewed attention to the overall need to consider sex when examining therapeutic outcomes for *any* treatment, particularly in neonatal populations where sex is often ignored. Notably, this routine failure to consider neonatal sex persists despite historic cases where clinicians overlooked evidence of sex to the detriment of one sex. An important example is indomethacin given to preterm infants to reduce the risk of intraventricular hemorrhage. Evidence came to light almost ten years after the original publication that prophylactic indomethacin treatment selectively benefited male infants [48] and showed little to no benefit for female infants, despite the risk of side effects [49]. Yet indomethacin continues to be widely administered to at-risk neonates without regard to sex [50,51]. Similar sex differences have recently come to light with regard to hypothermia treatment. Substantial evidence from animal research shows that neonatal hypothermia for HI may benefit females more than males, a difference that can be detected when large sample sizes are used [26,29]. Yet all of the large-scale neonatal hypothermia studies have routinely failed to report outcomes as a function of sex, precluding even the use of literature-based meta-analysis to evaluate sex across studies that did not individually reach significance [52]. Of note is a re-analysis of one small study of hypothermia that examined the effect of sex but was underpowered for such an analysis [53]. 

In closing, we note several limitations to our study. As a retrospective chart review, we cannot ascertain the physician-specific decision processes to determine which mothers received MagSul, nor do we have access to the patient-specific drug brand, composition or dosage. Being administered to the mother and not directly to the infant, this was not noted in infant medical records (beyond that MagSul was used). We do note that consistency of supplier and concentration was likely given that our patients were all born in a single facility. We also do not have access to information about the parent’s socioeconomic status or education level, which can greatly influence early cognitive and language outcomes. Future studies should make an effort to gather this information to generate a more complete picture. Finally, we acknowledge the limitations of performing a single-center study. Although it does offer greater consistency in some variables (e.g., common practices in treatment and care), it also limits us to a regionally homogeneous population. Future replications that include data from different institutions would help to extend our results to a broader racial, ethnic, and socioeconomic population, and are certainly needed. Other possible limitations, such as changes in clinical practice over the study time period and health conditions of the subjects, have been addressed to our best ability methodologically, but could be even further controlled by the collection of additional data such as detailed delivery, intervention, and health statistic parameters. 

## 4. Materials and Methods

All methods were approved by the applicable Institutional Review Board (IRB). Connecticut Children’s Medical Center IRB #19-112 and The University of Connecticut Health Center IRB 19X-211-1. 

### 4.1. Subjects

Subjects were infants admitted to and cared for at the University of Connecticut Health Center (UCHC)/Connecticut Children’s NICU at UCONN Health between 1 January 1991 and 31 December 2017. All subjects were born at 23–30 weeks GA, did not have an IVH/PVH rated greater than grade 3, and had received caffeine or theophylline. Ninety-one percent of the infants were treated with an adenosine antagonist, forming the base sample population (n = 858). The 81% who also had an ~18-month neurodevelopmental follow-up were retained (n = 696) and formed the final study population. Infants who did not receive caffeine or theophylline were not included in the study population, as this study population was originally collected for a study on the timing of xanthine (caffeine or theophylline) treatment [34]. Additionally, these infants were on average healthier, as defined by greater GA, higher birthweight, and shorter hospital stays, justifying their exclusion from the general study population. 

### 4.2. Data Collection

Data were collected and stored in a computerized database, *Neonatal Information System 3* (Medical Data Systems, Phil, PA, USA). Data from all patients admitted to the University of Connecticut Health Center (UCHC) or Connecticut Children’s NICU at UCONN Health from 1 January 1991 to 31 December 2017 who matched inclusion criteria were collected. We divided our data into three-decade groups to help account for changes in standard of care: 1991–2000, 2001–2010, and 2011–2017. The data were originally collected for clinical purposes. These data were collected and entered into the data base by trained NICU nurses. Only the variables that were of interest for this project were collected from this original clinical data (GA, preeclampsia, chorioamnionitis, magnesium sulfate treatment, mode of delivery, birth year, sex, birth weight, Apgar score, cord pH, Snap score, IVH rating, length of stay, type of xanthine treatment, and length of treatment) (same method and dataset used in [34]). NICU data nurses were responsible for collecting the original data and used strict definitions for each variable. Variable definitions did not change over the study time period. As this was a retrospective study, consent was not obtained. Subjects were deidentified at the time of data collection, and no identifiable information was collected; subjects were assigned a new subject number (see [34]). 

As far as the authors know, the brand, dosage, and composition of the MagSul administered in the UCHC was consistent over the period of the study. We do not, however, have specific records as we could only collect information from subject records and not maternal medical records. 

Behavioral and cognitive outcomes for the infants came from follow-up visits at UCHC or Connecticut Children’s Medical Center (CCMC) obtained at 18 months of age (corrected to GA at birth). Follow-up data were collected and stored in the *High-Risk Follow-Up* section of the NIS database and the CCMC NICU *Neurodevelopmental Follow-up Clinic database*. Outcomes were measured using the Bayley II and III or Cognitive Adaptive Test/Clinical Linguistic and Auditory Milestone Scale (CAT/CLAMS) assessments. Only infants who had at least cognitive and language outcome measures were included in this study (81% retention from the base sample, final n = 696; see [34]). Multiple editions of the Bayley Scales were available during the study time period (Bayley II–III). There were infants who received the Bayley III (n = 175), the Bayley II (n = 97), the CAT/CLAMS (n = 413), and both the Bayley II and CAT/CLAMS (n = 11). Then, we proceeded with Z-scoring of relevant sub-scale measures that were then averaged together into domain areas (language and cognition).

Individual scores were split into their respective testing components. For the Bayley, this included Gross Motor, Fine Motor, Problem Solving, Expressive Language, Receptive Language, Language Articulation, Self-Help, and Relationships with Others; for the CAT/CLAMS (the Cognitive Adaptive Test (CAT) and the Clinical Linguistic Auditory Milestone Scale (CLAMS)), sub-components included Cognitive and Language scores. Extraction of sub-scores was necessary because not all subjects received all tests (due to infant drop-out, non-participation during the exam, etc.), resulting in many partial profiles. Raw scores from all sub-tests were converted to z-scores for each subject, using the formula z = (x − μ)/σ, where x is the raw score, μ is the population mean, and σ is the population standard deviation. This calculation was performed independently for each assessment sub-test within each of the three decades (1991–2000, 2001–2010, and 2011–2017), accounting for changes in practices and/or testing over the 27 years. These sub-test Z-scores were sorted into four categories or domains, though only two are reported in this study—Social (comprised of the Self-help and Relationship with others Z-scores), Cognitive (comprised of Problem Solving, and the CAT Z-scores), Language (comprised of Expressive Language, Receptive Language, Language Articulation, and the CLAMS Z-Scores), and Motor (comprised of Gross Motor and Fine Motor Z-scores). An average z-score was obtained for each domain for each subject after all of the sub-tests had been Z-scored for each subject, by decade.

Use of the data in this way was necessary as not all subjects could complete all of the testing tasks, and not all had scores for all of the assessments. This allowed us to utilize a larger group of subjects who had received some cognitive or language assessment on either the Bayley or Cat/CLAMS. However, few subjects completed testing that allowed to calculate their motor and social domain z-scores. This is why we only report the cognitive and language outcomes. Our Z-scoring process allowed us to standardize individual performance without bias to a particular group of infants. The literature also shows that both Bayley and CAT/CLAMS have highly inter-correlated measures, which supports the use of the data in this way [54]. 

### 4.3. Statistical Analysis

After the data was collected, de-identified, and Z-scored, it was entered into *SPSS* 28 (IMB) for analysis. Initial assessments were performed using several health indices (gestational age, Apgar score, and length of NICU stay) to evaluate comparability of the groups that did and did not receive MagSul using a Multivariate ANOVA. This same assessment was also performed by sex. We also looked at the relationship between sex and reason for delivery, namely preeclampsia and premature rupture of membranes, by running a Pearson Chi-Square to see if the categorical variables were related. Next, the overall effect of sex on outcomes was examined using a multivariate ANOVA (sex as a between-variable). Subsequently, interactions between sex and magnesium sulfate (MagSul) treatment (y/n) were assessed, again using an ANCOVA test (sex and MagSul treatment as between-variables), with decade of birth and presence of preeclampsia included as covariates. These covariates were included to control for potential changes in medical practice over time, though we did not see any significant difference in outcome by decade, as well as to determine benefits from the specific use of MagSul to treat maternal eclampsia (which can by itself lead to poorer infant outcomes). Subsequently, simple effects ANCOVAs were run to identify specific group differences (between male MagSul, male no MagSul, female MagSul, and female no MagSul), again using decade of birth and preeclampsia as covariates.

## 5. Conclusions

In conclusion, although MagSul plays an important role in mitigating maternal seizures and death when there is risk of eclampsia—and clinical benefits of MagSul clearly outweigh risks in these cases—our results show that the routine use of MagSul in mothers who are *not* at risk of eclampsia needs far more consideration. Our results show that cognitive and language outcomes for male infants at 18 months are significantly *reduced* by the antenatal use of MagSul. Further studies on the impact of perinatal MagSul treatment should be performed using sex as a variable, but in the interim, our results clearly call into question assumptions that this drug treatment is widely beneficial in the absence of pre-eclampsia risk. Our cautionary findings extend further to many treatments used in preterm infants, including hypothermia, caffeine, and other neonatal interventions that may have more subtle benefits beyond mortality and CP [25,26,29,48,49,50]. In general, increased attention to the role of sex differences in clinical treatment of the neonatal population is warranted, given evidence that some null or minimal effect-size findings could be obscured by underlying sex differences that are currently often ignored. With specific regard to MagSul, additional research is needed to confirm these reported deleterious effects on male infant outcomes in order to ensure the best care for the at-risk newborn population. 

## Figures and Tables

**Table 1:** Means, standard error, and ANOVA significance for health indices as a function of MagSul group.

Measure	Group	Mean	SE	Significance
Weight (g)	No MagSul	1044.36	20.02	*p* > 0.05
	MagSul	1001.87	14.06	
Gestational age (Weeks)	No MagSul	27.43	0.12	*p* > 0.05
	MagSul	27.14	0.09	
Length of Stay (Days)	No MagSul	83.08	1.88	*p* > 0.05
	MagSul	85.21	1.49	
Apgar Score (5 min)	No MagSul	7.55	0.10	*p* > 0.05
	MagSul	7.70	0.07	

**Table 2:** Means, standard error, and ANOVA significance of health indices by sex.

Measure	Group	Mean	SE	Significance
Weight (g)	Female	934.28	37.95	*p* = 0.014
	Male	1058.88	33.46	
Gestational Age (Weeks)	Female	26.61	0.23	*p* > 0.05
	Male	27.06	0.21	
Length of Stay (Days)	Female	90.49	3.45	*p* > 0.05
	Male	89.13	2.35	
Apgar Score (5 min)	Female	7.69	0.08	*p* > 0.05
	Male	7.60	0.09	

**Table 3:** Frequency of different causes of premature delivery and Chi-Square results by sex.

Reason for Premature Delivery	Sex	n	Significance
Preeclampsia	Female	68	*p* > 0.05
	Male	52	
Premature Rupture of Membranes	Female	103	*p* > 0.05
	Male	110	

## Data Availability

The datasets presented in this article are not readily available because of privacy concerns, requests to access the datasets should be directed to the corresponding author.

## Abbreviations

MagSul = Magnesium Sulfate; CP = cerebral palsy; NMDA = N-methyl-d-aspartate; GM/IVH = germinal matrix or intraventricular hemorrhage; AA = Adenosine antagonist, GA = Gestational Age; H I= Hypoxia Ischemia
